# Isolation and characterization of the midgut microbiota of *Aedes albopictus* to identify suitable candidates for paratransgenesis

**DOI:** 10.1093/eurpub/ckae110

**Published:** 2025-01-13

**Authors:** Mersa Darbandsari, Majid Asgari, Mohammad R Abaei, Zahra Ghorbanzadeh, Maryam Derikvand, Patrick Okwarah, Navid Dinparast Djadid, Abbasali Raz

**Affiliations:** Malaria and Vector Research Group (MVRG), Biotechnology Research Center (BRC), Pasteur Institute of Iran, Tehran, Iran; Department of Biotechnology, Islamic Azad University, Tehran Medical Branch, Tehran, Iran; Malaria and Vector Research Group (MVRG), Biotechnology Research Center (BRC), Pasteur Institute of Iran, Tehran, Iran; Department of Medical Entomology and Vector Control, School of Public Health, Tehran University of Medical Sciences, Tehran, Iran; Malaria and Vector Research Group (MVRG), Biotechnology Research Center (BRC), Pasteur Institute of Iran, Tehran, Iran; Malaria and Vector Research Group (MVRG), Biotechnology Research Center (BRC), Pasteur Institute of Iran, Tehran, Iran; Infectious Hazard Prevention and Preparedness Unit, Department of Health Emergency, World Health Organization, Eastern Mediterranean Regional Office, Cairo, Egypt; Department of Community Health, Amref International University, Nairobi, Kenya; Malaria and Vector Research Group (MVRG), Biotechnology Research Center (BRC), Pasteur Institute of Iran, Tehran, Iran; Malaria and Vector Research Group (MVRG), Biotechnology Research Center (BRC), Pasteur Institute of Iran, Tehran, Iran

## Abstract

*Aedes albopictus* is a widely recognized carrier of various pathogens. Its resilient characteristics enable it to easily spread across diverse climates. The microbiota in the midgut of mosquitoes plays a crucial role in the interactions between the host and pathogens and can either enhance or reduce the ability of the insect to transmit diseases. Hence, determining the microorganisms present in the mosquito’s digestive system could be a promising approach to developing an effective method of controlling them. Hence, the aim of this study was to investigate the microbial compositions in the midguts of *Ae. albopictus* mosquitoes collected from the fields of Sistan and Baluchestan Province. The midguts of 60 female mosquitoes were dissected, and their related bacteria were determined using the culture-dependent method. Different colonies were differentiated using the biochemical tests followed by 16S rRNA gene sequencing. The isolated bacteria were identified as belonging to the *Asaia*, *Delftia*, *Serratia*, *Aeromonas*, *Paracoccus*, and *Planomicrobium* genera based on biochemical and molecular analysis. The findings obtained in this study were largely consistent with earlier studies conducted on mosquitoes gathered from different regions throughout the world. Overall, the findings could enhance our understanding of the microbial diversity in *Ae. albopictus* and aid in the identification of a potent and widespread bacterium for the development of a paratransgenesis tool to combat *Aedes*-borne infectious diseases.

Key points

- Isolation of the symbiotic microbiota from the midgut of *Ae. albopictus* using culture-dependent methods.
- Identification of the isolated bacteria using biochemical and molecular techniques.
- Characterizing the bacterial symbionts present in the midgut of *Ae. albopictus* mosquitoes collected from the field.
- Selecting a bacteria to develop the paratransgenesis strategy for combating *Aedes*-related infectious diseases.

## Introduction

Biological invasion of alien species is a global issue that threatens the health and economy of different countries [1], complicated by new technologies and transportation systems. Vector-borne diseases are one of these issues that are broad in scope and significant, especially in the new world. Concerning pathogen transmission, it also has adverse impacts on human and animal well-being [2]. Mosquitoes are one of the biological agents that threaten our community, have a potential role in disease transmission, have good adaptation ability to new conditions, and could be transferred among the countries [3]. The Asian tiger mosquito, *Aedes (Stegomyia) albopictus* [4], is native to the South-East of Asia and is considered the most highly invasive species in the world [5, 6]. Besides, *Ae. albopictus* is an active vector of at least 26 harmful types of arboviruses, such as Chikungunya, Zika, and Dengue viruses, which are adversely threatening millions of people around the world [6, 7]. Based on the World Health Organization report, 40% of people who live in more than one hundred countries, mainly in Southeast Asia, and some regions of the Western Pacific and America, are affected by dengue fever [8]. It seems that, among the different approaches, vector control is more effective and applicable for preventing arboviral diseases [9]. Due to the invasive nature of the mosquito species, surveillance and control are the fundamental strategies that should be implemented at national and local levels [10]. Considerably, there are different challenges regarding conventional vector-control methods, such as insecticides, biological agents, and environmental sanitation [11].

Recent studies on mosquitoes revealed that diverse groups of microbiota are inside the mosquito midgut that share a symbiotic relationship with various impacts and benefits [12–14]. Yadav and colleagues isolated phylum *Bacteroidetes* from the collected *Ae. albopictus* in Arunachal Pradesh, India [9]. It is also well established that mosquitoes’ microbiota compositions could significantly affect their ability for disease transmission [13, 15]. Therefore, genetic manipulation of the midgut microbiota could be a promising strategy to develop novel and robust vector control tools. This technology is known as paratransgenesis. Indeed, isolating and characterizing the mosquito microbiota is a prerequisite for this technology.

As an alternative strategy for controlling the *Aedes* mosquitos, paratransgenesis has recently caught scientists’ attention. The base of this strategy is the genetic manipulation of endosymbionts to express antipathogen effector molecules inside the target host [16, 17]. Thus, understanding mosquito microbiota is imperative for developing a bacterium-based paratransgenic tool. Therefore, the main objective of this study was to assess the midgut microbiota of *Ae. albopictus* using biochemical and molecular approaches to find and introduce potent paratransgenesis candidate bacteria and provide the fundamental data for selecting the best bacterium for future steps.

## Methods

### Samples collection and midgut dissection

Adult mosquitoes were caught from the Beris village of Sistan and Baluchestan Province from South-East of Iran. Then samples were transferred to the national insectary of the Pasteur Institute of Iran and maintained inside a quarantined room (26–28°C, 60%–80% humidity, 12/12 light and darkness conditions). The adult mosquitoes were morpho-taxonomically identified as described by Darsie and Samanidou-Voyadjoglou [18].

In order to evaluate the midgut microbiota, a total of 20 adult female mosquitoes were carefully dissected in isolated and sterile conditions. To this end, all adult mosquitoes were anesthetized at 20°C for 3 min. Then, samples were surface sterilized by immersing them in 75% ethanol, followed by washing them twice with sterile phosphate-buffered saline (PBS). After individually dissecting, the midguts were transferred into the sterile 1.5 ml microtube, and then 100 μl of PBS was added to the tube and homogenized with a sterilized micropestle. Prior to dissection, all the applied apparatus were sterilized using 75% ethanol. Morpho-taxonomic identification of adult mosquitoes was done as described [2].

A total of 60 adult female mosquitoes were carefully dissected in isolated and sterile conditions to evaluate the midgut microbiota. To this end, all adult mosquitoes were anesthetized at −20°C for 3 min. Sterilization of the surface of the samples was performed by immersing them in 70% ethanol, followed by twice washing with PBS. After individually dissecting, each midgut was transferred into a 1.5 ml sterilized microtube, and then 100 μl of PBS was added to the tube and homogenized with a sterilized micropestle. Before and after each dissection, all the applied apparatus were sterilized using 70% ethanol and flame.

### Midgut bacteria isolation and microbial characterization

All media were purchased from HiMedia Co. (India). The isolated midgut content was divided into two different equal suspensions, i.e. Luria-Bertani (LB) and *Asaia* broth media. The suspension of homogenized midguts was plated on three agar media, including LB agar, MacConkey agar, and *Asaia* agar, each suitable for Gram-positive, Gram-negative, and *Asaia* bacteria growth, respectively. The LB agar and MacConkey agar plates were incubated at 37°C overnight; additionally, the *Asaia* agar plates were incubated at 30°C for three days. After that, the obtained bacterial colonies on the plates were individually differentiated based on morphological features such as shape, size, and colour. To reach pure colonies, the distinguished colonies were chosen for subculturing on the appropriate agar plates. Determining the gram type was also carried out on bacterial colonies using the gram staining method. First, the isolated colonies were fixed on a clean glass slide by passing through a burner’s flame. The smear was stained with crystal violet and maintained for 50 s. The slide was then washed with distilled water, flooded with Lugol’s solution, kept for 1 min, and washed off with water. The slides were thoroughly covered by acetone-alcohol for 20 s and washed off with water immediately. Finally, the counters were stained with safranin for 20 s. Afterward, the slides were air-dried and observed under a microscope with an oil-immersion lens (100X magnification).

After homogenization, the suspension of midgut content was divided into two equal volumes and inoculated into the brain–heart infusion (BHI) and *Asaia* broth media. Then, the BHI broth and *Asaia* broth media were incubated at 37°C overnight and 30°C for three days, respectively. For isolating the grown bacteria in broth media, 50 ml of the BHI broth medium was plated on LB agar and MacConkey agar from the BHI broth medium, and *Asaia* agar from the *Asaia* broth medium.

The LB agar and MacConkey agar plates were incubated at 37°C overnight and the *Asaia* agar plates at 30°C for three days. Thereafter, the obtained bacterial colonies on the plates were individually differentiated based on morphological features such as shape, size, color, and halo zone around the colonies (especially for *Asaia* sp.). To isolate the pure colonies, the distinct colonies were chosen for subculturing on the appropriate agar plates. Determining the gram type was also carried out on bacterial colonies using the gram staining method. The isolated colonies were fixed on a clean glass slide by passing through a burner’s flame. Then, the smear was stained with crystal violet and maintained for 50 s. Next, the slide was washed with distilled water, flooded with Lugol’s solution, kept for 1 min, and washed off with water. Then, the slides were covered by acetone-alcohol for 20 s thoroughly, washed off with water immediately, and counter bacteria were stained with the Safranin for 20 s. Afterward, the slides were air-dried and observed under a light microscope with the oil immersion lens (100X magnification).

### Biochemical characterization

In addition to morphological differentiation, biochemical approaches were used for bacterial population differentiation. Therefore, citrate utilization, urease, oxidase, sulfur indole motility media, triple sugar iron, and mannitol biochemical tests were done for Gram-negative bacteria, and staining with crystal violet was performed for Gram-positive bacteria. All materials were purchased from HiMedia Co. (India). Bacteria with morphological and biochemical differences were selected for molecular analysis, sequencing of the 16srRNA gene, and nucleotide Basic Local Alignment Search Tool (BLAST) analysis against the 16 s ribosomal RNA sequences (Bacteria and Archaea) (rRNA and ITS databases) [19].

### Genomic DNA extraction and polymerase chain reaction amplification of 16 s rRNA gene

Genomic DNA was extracted from the bacterial pellets obtained from the LB broth medium using the MBST DNA extraction kit (Iran) following the manufacturer’s instructions. The final elution volume was 30 µl (TE buffer). The quantity and quality of the extracted DNA were evaluated using 0.8% agarose gel electrophoresis and spectrophotometry (Titertek Berthold, Bad Wildbad, Germany), respectively. The 16S-F (5′-AGTTTGATCCTGGCTCAG-3′) and 16S-R (5′-GCTACCTTGTTACGACTTC-3′) primer pair which amplifies the 16S rRNA gene was used as forward and reverse primers, respectively [20]. Moreover, Asafor (5′-TGGCGGACGGGTGAGTATC-3′) and Asarev (5′-GGCTTCGGGTCAAACCAACT-3′) were used as specific 16S rRNA gene primers for confirming the *Asaia* sp. samples [19]. Each reaction included 400 nM of each primer, 1.5 mM MgCl_2_, 1 unit of Taq DNA polymerase (Cinaclon, Iran), 0.2 mM dNTPs, 2.5 µl of 10X reaction buffer, and 150 ng of genomic DNA as the template. The polymerase chain reaction (PCR) assay was carried out by an initial denaturation at 95°C for 5 min, followed by 35 cycles of 95°C for 30 s, 60°C for 30 s, 72°C for 60 s, followed by a final extension at 72°C for 10 min. The amplicons were visualized on 1.2% agarose gel by staining with Safe Stain (Cinaclon, Iran).

### TA-cloning and sequencing

After confirming the expected length of the 16S rRNA amplicon, a library of the 16S rRNA gene was constructed by ligating the target gene amplicon into the pTG19-T vector (Vivantis, Malaysia) according to the manufacturer’s instructions. Afterward, the ligation products were transformed into the *E. coli DH5α* through the heat shock transformation method. Then, the transformed bacteria were plated on the LB-Agar plates containing ampicillin (50 μg/mL) (Sigma-Aldrich, USA), X-gal (100 mM) (Sigma-Aldrich, USA), and Isopropyl β-D-1-thiogalactopyranoside (100 mM) (Sigma-Aldrich, USA). Among the grown colonies, positive clones were primarily selected based on the morphological features of the colonies (white color). After culturing in the LB-broth media, related plasmids were extracted from the positive colonies by the plasmid extraction kit (GeneAll, South Korea). Moreover, further validation was done by PCR amplification with the gene-specific primers, and amplicons were visualized using agarose gel electrophoresis.

The obtained and confirmed plasmids were directly sequenced on an ABI 3500xl Genetic Analyzer (Applied Biosystems Inc. Foster City, CA) from both plus and minus strands using the M13 forward and M13 reverse primers (Bioneer, South Korea). The obtained sequences were checked and analysed using the Chromas Lite software (ver. 2.1). Afterward, sequences were analysed using the web-based nucleotide BLAST software (https://blast.ncbi.nlm.nih.gov/blast/Blast.cgi) to search the homologous sequences.

### Bioinformatics analysis

The obtained sequences in this study were analysed as the query in the NCBI database using the nucleotide BLAST tool [21] for searching the homologous sequences. This homology search was performed to confirm the biochemical test results. In the next step, the phylogenetic relatedness of the sequences was determined using the Molecular Evolutionary Genetics Analysis (MEGA) software (Ver. 7.0) with 1000 ultrafast bootstrap replicates as default parameters. The phylogenetic tree was reconstructed using the Neighbour-Joining (NJ) method. The human 18S rRNA gene (GenBank accession number: M10098.1) was used as an outgroup for phylogenetic tree construction.

## Results

### Isolation of bacteria and biochemical analysis of the isolates

**Figure 1.**
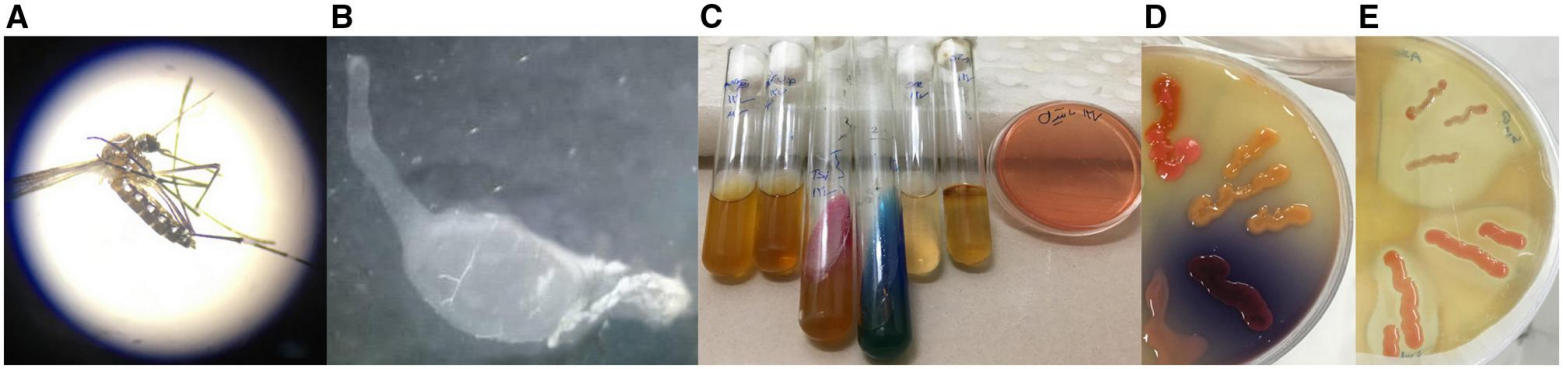
Different steps of *Ae. albopictus* bacteria isolation. (A) Morphological features evaluation and confirmation, (B) dissection of the mosquito midgut, (C) biochemical tests for differentiation of the isolated bacteria, and (D, E) isolated *Asaia* sp. colonies on *Asaia* agar.

**Figure 2.**
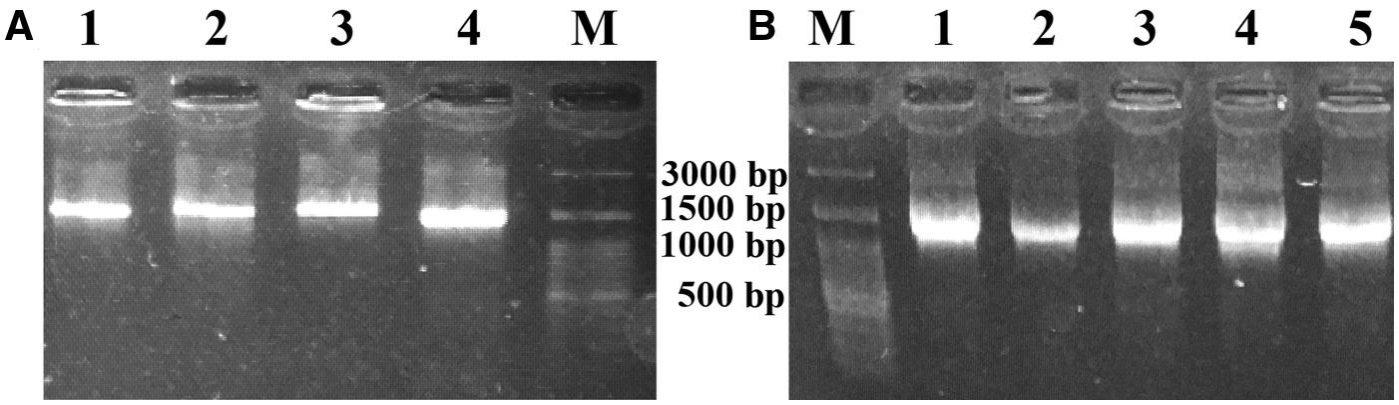
Phylogenetic tree of the isolated bacteria from the midgut of *Ae. Albopictus*.

**Figure 3.**
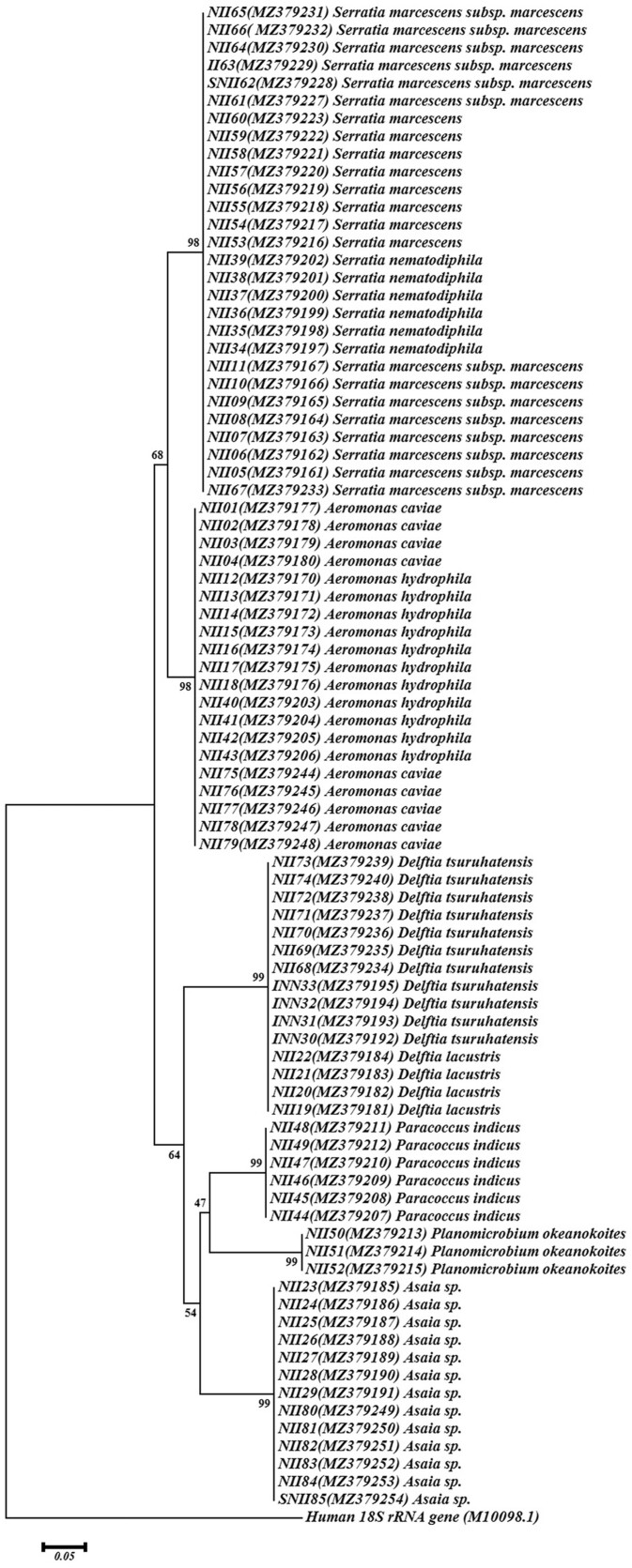
Agarose gel electrophoresis of 16S rRNA amplicons. (A) 1–4 lanes are related to the 16S rRNA gene amplification of different isolated colonies, and M is 100 bp DNA marker; (B) 1–5 lanes are related to different isolated *Asaia* sp. colonies, and M is 100 bp DNA marker.

In this study, the diversity of midgut microbiota from the field-collected *Ae. albopictus* mosquitoes was evaluated. The first step was to evaluate the morphological features of the selected mosquitoes and confirm their genus and species. Analysis of the microbiota of 60 mosquito midguts was performed. According to the morphological feature, 153 colonies were selected for biochemical analysis, and finally, 85 colonies were chosen for molecular analysis (Fig. 1). Amplicons of the 16S-F/16S-R and Asafor/Asarev primer pairs were 1500 bp and 1200 bp, respectively (Fig. 2A and B). After TA-cloning and sequencing, all the acquired sequences were submitted to GenBank (Fig. 3). According to the acquired results, the isolated bacteria mainly belonged to the Gram-negative bacteria (Table 1).

**Table 1.** Isolated bacteria from the midgut of *Ae. albopictus* mosquitoes by culture-dependent method

No.	Name	Phylum	Gram stain
1	*Aeromonas caviae*	Proteobacteria	Gram-negative
2	*Aeromonas hydrophila*	Proteobacteria	Gram-negative
3	*Delftia tsuruhatensis*	Proteobacteria	Gram-negative
4	*Delftia lacustris*	Proteobacteria	Gram-negative
5	*Paracoccus indicus*	Proteobacteria	Gram-negative
6	*Planomicrobium okeanokoites*	Firmicutes	Gram-positive
7	*Serratia marcescens subsp. marcescens*	Proteobacteria	Gram-negative
8	*Serratia nematodiphila*	Proteobacteria	Gram-negative
9	*Serratia marcescens*	Proteobacteria	Gram-negative
10	*Asaia* sp.	Proteobacteria	Gram-negative

The evolutionary history was inferred using the NJ method. The optimal tree with the sum of branch length = 1.04827900 is shown (Fig. 2). The percentage of replicate trees in which the associated taxa clustered together in the bootstrap test (1000 replicates) is shown next to the branches. The tree is drawn to scale, with branch lengths in the same units as those of the evolutionary distances used to infer the phylogenetic tree. The evolutionary distances were computed using the maximum composite likelihood method and are in the units of the number of base substitutions per site. The analysis involved 86 nucleotide sequences. All positions containing gaps and missing data were eliminated. There were a total of 153 positions in the final dataset. Evolutionary analyses were conducted in MEGA7.0. The phylogenetic tree was constructed based on the 16S ribosomal RNA sequences. The human 18S rRNA gene (GenBank accession number: M10098.1) was used as an outgroup for phylogenetic tree construction. The accession number and the genus and species of each sample are shown in front of the sample number.

### Molecular analysis

Based on the morphological and biochemical characteristics of the colonies, the 16S rRNA gene sequence of the 85 isolates was analysed. The pair-ended sequencing of the 16S rRNA indicated the diversity of the midgut bacterial populations. The names of the identified bacteria are presented in Table 1. According to our results, the identified strains belong to the *Aeromonas* (*A. caviae* and *A. hydrophila*), *Delftia* (*D. tsuruhatensis* and *D. lacustris*), *Paracoccus*, *Planomicrobium*, *Serratia*, and *Asaia* genera. The 16S rRNA gene sequences of the targeted colonies were aligned to evaluate the relatedness of the isolated bacteria.

### Phylogenetic tree analysis

According to the phylogenetic tree analysis and among the identified bacteria, *Serratia* and *Aeromonas* genera have a close relationship; *Delftia*, *Paracoccus*, *Planomicrobium*, and *Asaia* genera are more related (Fig. 2).

## Discussion

*Ae. albopictus* is a known vector of medical importance, responsible for the spread and transmission of arboviruses worldwide [6]. It has also been reported that this species possesses a durable nature and easily copes with a broad temperature range and environmental situations, thus rapidly expanding its global territory [5, 7]. Consequently, continually expanding and spreading mosquitoes increasingly enhances the probability of new future disease outbreaks [22]. Therefore, providing new insights into the potential factors that could influence mosquitoes is vital. One of the main factors that affect the potential of disease transmission in mosquitoes is the composition of the midgut microbiota, as they play an essential role in the host-pathogen and host-symbiont interactions [10, 14].

Paratransgenesis has been introduced as a promising strategy for controlling vector-borne diseases recently [23]. Hence, the diversity of midgut microflora from the field-collected *Ae. albopictus* was evaluated by implementing the morphological, biochemical, and molecular approaches in this study to improve our knowledge and prepare the prerequisite data for building the blocks of future studies.

In this study, 60 adult females of *Ae. albopictus* were collected and verified based on the morphological keys. Among isolated bacteria, 153 and 85 colonies were selected for biochemical tests and molecular analysis, respectively. The molecular results revealed that most of them belong to gram-negative bacteria. Minard *et al.* [12] reported that gram-negative bacteria constitute a large percentage of the gut bacteria from *Ae. aegypti*, which is responsible for dengue fever, and the findings of this study are consistent with their results [13]. It is also worth noting that *Ae. albopictus* has been observed and recorded in the South East of Iran, where the first record of dengue fever was reported [13].

Moreover, further investigation of the isolated bacteria was carried out, and analysis of the nucleotide variations in their 16S rRNA sequences of 85 colonies revealed that those are related to the *Serratia*, *Aeromonas*, *Delftia*, *Paracoccus*, *Planomicrobium*, and *Asaia* genera. Except for the *Planomicrobium okeanokoites* that is gram-positive and belong to the *Planococcaceae* family, other isolated bacteria are gram-negative and belong to the *Proteobacteria* phylum. Most of the identified bacteria in this study had been previously reported from the midgut of *Aedes* spp. and other mosquito species. The *Serratia*, *Aeromonas*, *Delftia*, and *Paracoccus* genera are known bacterial populations already detected from the mosquitoes’ gut and midgut worldwide [15, 17, 24].

It is well established that the susceptibility of chikungunya and dengue viruses is affected by bacterial compositions of the midgut microbiota of mosquitoes [25]. For instance, the presence of *Serratia odorifera* in *Ae. aegypti* midgut significantly enhances the susceptibility of *Ae. aegypti* for chikungunya virus. This is due to the interaction between the bacterial P40 protein and the gut membrane, which suppresses the immune response of *Ae. Aegypti* [16, 18]. In addition, uncovering the genetic composition of the mosquitoes’ midgut will open up new windows toward finding and developing a paratransgenesis approach against the *Ae. Aegypti*, which is considered an invasive mosquito [26]. To our knowledge, this is the first report of *Delftia lacustris* presence in *Ae. albopictus*, whose isolation has been reported in *An. gambiae* previously [27]. Nevertheless, other bacterial strains belonging to the *Delftia* genus have already been reported from *Aedes* mosquitoes [17, 24]. Therefore, evaluating the properties of *Delftia lacustris* in future studies could determine its potential abilities as a candidate agent for paratransgenesis.

In addition to known gram-negative and gram-positive bacteria, a bacterial population belonging to *Asaia* sp. was isolated. This bacterium is a symbiont of different mosquito species; additionally, it is a potential candidate for blocking the transmission of mosquito-borne pathogens through paratransgenesis [28]. Indeed, due to having various features such as conserved association with the host insect, easy cultivation and genetic manipulation with the exogenous DNA, and transovarial and transstadial transmission routes, *Asaia* is a serious candidate for vector control strategies [29]. Successful investigations of *Asaia* sp. in mosquitoes are mainly confined to the *Anopheles* species. Recently, Rosso *et al.* successfully modified and transformed an isolated strain of *Asaia* from *Anopheles stephensi* to secrete anti-Plasmodium molecules against *Plasmodium berghei* [30]. Furthermore, Cappelli *et al.* [31] reported that the introduction of *Asaia* isolates not only enhances the immune responses but also reduces the development of malaria parasite oocysts in *An. stephensi*. In 2015, Bongio and Lampe [32] performed a study on the *Asaia* SF2.1 strain and identified new secretion signals that could be used to secrete biomolecules against the pathogen or vector by this bacterium. In 2020, Asgari *et al.* [33] characterized the RNase III enzyme of *Asaia* sp. to create a potential tool for using *Asaia* bacterium for dsRNA production and knocking down the target genes. Nevertheless, no published evidence of using *Asaia* sp. for controlling the *Aedes* species has been reported yet, and regarding its potential capabilities and isolation in this study; this bacterium could be considered as a dormant candidate for combatting against *Aedes*-borne diseases.

Moreover, *Serratia* sp. has been identified in *Aedes* sp. in different studies as a symbiont bacterium [11]. This species has good features for application in paratransgenesis, and most of the studies performed as a paratransgenesis agent have been done on *Anopheles* species [34]. In this study, *Serratia marcescens* and *Serratia nematodiphila* were isolated from the dissected samples. In 2010, Gusmão *et al.* [35] isolated *Serratia* sp. from the midgut of *Ae. aegypti* by culture-dependent and independent methods. They reported that the genus *Serratia* was the dominant microorganism, and its frequency was 54.5% of the total isolated bacteria. In 2021, Alvarado *et al.* [36] isolated *Serratia* sp. from the ovary of *Ae. aegypti* by culture-dependent methods. Besides, different studies on *Serratia* species and paratransgenesis have been done on other medically important vectors.

The work employed a culture-dependent method to characterize the bacteria. However, the use of culture-independent and high-throughput technologies could enhance our comprehension of the variety of midgut microbiota in specific species [37, 38]. Both the culture-dependent and culture-independent techniques possess distinct advantages and disadvantages. One of the factors for selecting candidates for paratransgenesis is the ease of culturing them, a thorough understanding of their genomic maps, and the availability of genetic engineering technologies applicable to them. The majority of bacteria that are recognized using culture-dependent methods possess these characteristics, or they can be caught using advanced genetic techniques. In culture-independent approaches, the abundance of detected bacteria is enhanced, but a significant proportion of them are noncultivable, and their genetic manipulation is highly intricate. For instance, *Wolbachia* is employed in the control of *Aedes* spp., but its preparation for paratransgenesis requires advanced technology that is not accessible to the majority of the involved countries [37, 38].

The results of the current study are consistent with the previously performed studies in different regions of the world on field-collected samples and require further experimental investigations to evaluate the paratransgenic application of the suitably identified bacteria [37]. It has been approved that midgut microbiota composition has a potential role in vector competence and increasing or decreasing the vectorial capacity of mosquitoes [37]. Therefore, outstanding attention is required to develop an efficient controlling strategy to prevent any upcoming *Aedes*-borne arboviruses outbreaks. To sum up, we postulate that the presented results further shed light on the diversity of the midgut microbiota of *Ae. albopictus*, which could help select an effective symbiont microorganism to develop a paratransgenesis agent and decrease the vectorial capacity of mosquitoes and transmission of *Aedes*-borne diseases in future studies.

## Data Availability

The sequences obtained and/or analysed during the present study are deposited in the GenBank database under the following accession number: M10098.1.
